# Addressing the inequalities in global genetic studies for the advancement of Genetic Epidemiology

**DOI:** 10.3126/nje.v13i4.61271

**Published:** 2023-12-31

**Authors:** Brijesh Sathian, Edwin van Teijlingen, Bedanta Roy, Russel Kabir, Indrajit Banerjee, Padam Simkhada, Hanadi Al Hamad

**Affiliations:** 1 Geriatrics and long term care department, Rumailah Hospital, Hamad Medical Corporation, Doha, Qatar; 2 Centre for Midwifery& Women’s Health, Bournemouth University, Bournemouth, UK; 3 Department of Physiology, Faculty of Medicine, Quest International University, No. 227, Plaza Teh Teng Seng (Level 2), Jalan Raja Permaisuri Bainun, 30250 Ipoh, Perak Darul Ridzuan, Malaysia; 4 School of Allied Health, Anglia Ruskin University, Essex, UK; 5 Sir Seewoosagur Ramgoolam Medical College, Belle Rive, Mauritius; 6 School of Human and Health Sciences, University of Huddersfield, Huddersfield, UK

## Background

The human genome has evolved significantly since Watson and Crick’s 1953 discovery of DNA (deoxyribonucleic acid) structures [1]. Advances in RNA (ribonucleic acid) vaccines and recombinant DNA technology have made genetics accessible to the public [2]. In 2023, Katalin Karikó and Drew Weissman jointly received the Nobel Prize in Physiology or Medicine for their groundbreaking work in developing COVID-19 mRNA vaccines. Medical genetics began with single-gene disease inheritance patterns, and as of 2023, a total of 4,873 genes had been mapped. The most common disease is polygenic [3]. Advances in genome technologies have led to sequencing and genotyping of millions of human genomes. However, these studies were primarily based on European populations, limiting the benefits of genomic research to underrepresented populations. It is necessary to propose a roadmap to enhance inclusion and ensure equal health benefits of genomics [4].

The COVID-19 pandemic has highlighted the importance of global resilience and emergency preparedness, particularly in pathogen surveillance systems. The emergence of SARS-CoV-2 variants has highlighted the importance of genomic sequencing data for detecting and characterising pathogens. Since the pandemic, global recommendations have been made, advocating to strengthen countries’ genomic surveillance capabilities. To provide high-quality, timely, and relevant public health interventions within local to global surveillance systems, the Global Genomic Surveillance Strategy for Pathogens with Pandemic and Epidemic Potential 2022–2032 seeks to enhance and scale-up genomic monitoring [5].

Genetic research is often a secondary priority for funding in many countries; however, international institutions and research-intensive nations can help increase diversity. Funders should reconsider the restrictions faced by researchers in their countries. Collaboration in genetic research can offer diverse multidisciplinary expertise, grant writing experiences, and local knowledge. Building long-lasting partnerships is crucial for research funding. However, power imbalances and community reactions should also be considered. Capacity-building and data-sharing agreements can support local expertise and hence promote genomic research. Funds should prioritize sustainability to improve genomic study diversity. Researchers can contribute to local capacity enhancement by hiring students or researchers in case there is no dedicated funding for capacity building. Another consideration is supporting cohort-based genomic research which requires access to essential infrastructure components and alignment with legal, administrative, and ethical frameworks at institutional and national levels. Educational models that retain trained individuals are crucial for narrowing genomic study gaps for underrepresented populations by transferring local technology and knowledge.

Bioinformatics tools are crucial for research efficiency, data management, and analysis, as population-scale sequencing and data sharing have been made possible by advancements in next-generation sequencing technologies in human genetics. For large-scale data analytics, moving from an old-fashioned approach to a contemporary one delivers scalability, portability, and repeatability. Because of container technology, published findings can be replicated, enabling users to use the same program on different HPC (High Performance Computing) servers and computational settings. A workflow description language and engines are used in modern workflows to provide portability across various computational environments and scalability to accommodate growing computational resource sizes. Workflow engines on GCP (Google Cloud Platform) like Terra and Cromwell can execute workflows on local machines, cloud platforms, and batch job schedulers. Administrators must install container runtimes, such as a docker engine and singularity, for multi-platform use [6].

The human reference genome assembly has been available for two decades, and advancements in sequencing technology have enabled rapid whole-genome sequencing in single institutes. WGS (whole-genome sequencing) data analysis applications will enable large-scale data analysis on multi-clouds, integrate datasets with a population scale, and ensure the reproducibility of publications through modern workflow engines and scalability. In human genetics, expert-knowledge-driven approaches from medical and biological professionals and data-driven approaches from computer science applied to epidemiology, such as AI (artificial intelligence), are required for domain-specific downstream data interpretations. For reliable diagnostic, prognostic, and therapeutic tools, as well as generalized outcomes, genomic studies should involve a wide range of majority and minority populations. The field of genomics in medicine is entering a new era, and to increase the application of gene therapy in the treatment of emerging infections and disorders, there needs to be a united worldwide effort.

## References

[1] Watson JD Crick FH. The structure of DNA. Cold Spring Harb Symp Quant Biol. 1953;18:123–131. 13168976 10.1101/sqb.1953.018.01.020

[2] McKusick VA. Mendelian Inheritance in Man and its online version, OMIM. Am J Hum Genet. 2007;80:588–604. https://doi.org/10.1086/514346 10.1086/514346 PMid: ; PMCID: . 17357067 PMC1852721

[3] Johns Hopkins University. OMIM Gene Map Statistics. [online] 2023 [ cited 2023 Dec 22]. Available from: https://www.omim.org/statistics/geneMap

[4] Fatumo S Chikowore T Choudhury A Ayub M Martin AR Kuchenbaecker K. A roadmap to increase diversity in genomic studies. Nat Med. 2022 Feb;28(2):243-250. https://doi.org/10.1038/s41591-021-01672-4 Epub 2022 Feb 10. 10.1038/s41591-021-01672-4 PMID: ; PMCID: . 35145307 PMC7614889

[5] WHO. Global genomic surveillance strategy for pathogens with pandemic and epidemic potential 2022–2032: Progress report on the first year of implementation [Internet]. [online] 2022 [ cited 2023 Dec 22]. Available from: https://www.who.int/publications/i/item/global-genomic-surveillance-strategy-for-pathogens-with-pandemic-and-epidemic-potential-2022-2032--progress-report-on-the-first-year-of-implementation

[6] Tanjo T Kawai Y Tokunaga K Ogasawara O Nagasaki M. Practical guide for managing large-scale human genome data in research. J Hum Genet. 2021 Jan;66(1):39-52. https://doi.org/10.1038/s10038-020-00862-1 Epub 2020 Oct 23. 10.1038/s10038-020-00862-1 PMID:; PMCID: . 33097812 PMC7728600

